# Supplementary material for: Analysis of PPARγ Signaling Activity in Psoriasis
Source: Int J Mol Sci. 2021 Aug 10;22(16):8603. doi: 10.3390/ijms22168603 (PMC8395241; doi:10.3390/ijms22168603)
Supplement: Supplementary file 1 [file ijms-22-08603-s001.zip › Supplemental materials_Analysis of PPARg signaling activity in psoriasis/Pathway models/Models images and html files/Interleukin-17 and interleukin-22 signaling in psoriasis/3670.html]

HBEGF


# Protein HBEGF

|  |  |
| --- | --- |
| URN | urn:agi-llid:1839 |
| Total Entities | 0 |
| Connectivity | 2653 |
| Name | HBEGF |
| Description | heparin-binding EGF-like growth factor |
| Notes | activates epidermal growth factor receptor mediated signaling; contributes to neuronal survival; may play a role in neurogenesis during ventral midbrain development [RGD, Feb 2006] |
| Primary Cell Localization | Extracellular |
| Class | Ligand |

---

|  |  |
| --- | --- |
| Pathway | Platelet Activation via Adhesion Molecules |
|  | Proplatelet Maturation |
|  | Vascular Smooth Muscle Cell/Pericyte Migration and Proliferation |
|  | Vascular Endothelial Cell Activation by Blood Coagulation Factors |
|  | Proteins Involved in Atherosclerosis |
|  | Proteins Involved in Hypertrophic Cardiomyopathy |
|  | IL17 Signaling in Psoriasis |
|  | Proteins Involved in Psoriasis |
|  | Proteins Involved in Diabetic Nephropathy |
|  | Cetuximab Resistance in Colorectal Cancer |
|  | CagA Phosphorylation Dependent Signaling |
|  | Multiple Myeloma |
|  | Proteins Involved in Hepatocellular Carcinoma |
|  | Hepatocellular Carcinoma |
|  | Growth Factor Signaling in Hepatocellular Carcinoma |
|  | Pancreatic Ductal Carcinoma |
|  | Growth Factor Signaling in Pancreatic Neoplasms |
|  | Pancreatic Neuroendocrine Tumors |
|  | Proteins Involved in Breast Cancer Related to ERBB2/VEGFR/Akt Signaling Pathway |
|  | ERBB/VEGFR/Akt Signaling in Breast Cancer |
|  | ESR1/ERBB Positive Luminal Breast Cancer |
|  | Breast Cancer |
|  | Endometrial Cancer |
|  | Endometrioid Endometrial Cancer |
|  | Clear Cell Endometrial Cancer and Papillary Serous Endometrial Cancer |
|  | Endometrioid Ovarian Carcinoma |
|  | Proteins Involved in Endometrioid Ovarian Carcinoma |
|  | Proteins Involved in Mucinous Ovarian Carcinoma |
|  | EGFR and PTAFR Signaling in Ovarian Cancer |
|  | High-Grade Serous Ovarian Carcinoma |
|  | Low-Grade Serous Ovarian Carcinoma |
|  | Proteins Involved in Low-Grade Serous Ovarian Carcinoma |
|  | Ovarian Cancer |
|  | Mucinous Ovarian Carcinoma |
|  | Prostate Cancer |
|  | Cytokines Trigger Otitis Media |
|  | Elevated Receptors -> Expression Targets in Blood vessel |
|  | Elevated Receptors -> Expression Targets in Blood Vessel |
|  | EGF -> CTNN Expression Targets |
|  | EGF -> AP-1/ATF Expression Targets |
|  | EGF -> STAT Expression Targets |
|  | EGFR -> NCOR2 Signaling |
|  | EGFR/ERBB3 -> MEF/MYOD/NFATC/MYOG Signaling |
|  | EGFR -> CTNND Signaling |
|  | EGFR/ERBB2 -> TP53 Signaling |
|  | EGFR/ERBB2 -> CTNNB Signaling |
|  | ERBB2/3 -> EP300/ETS/ETV/SP1 Signaling |
|  | EGFR -> SMAD1 Signaling |
|  | EGFR/ERBB2 -> HIF1A Signaling |
|  | EGFR -> ZNF259 Signaling |
|  | EGFR -> AP-1/ATF2 Signaling |
|  | EGFR -> AP-1/CREB/ELK/SRF/MYC Signaling |
|  | EGFR/ERBB -> STAT Signaling |
|  | EREG -> EP300/SP1 Expression Targets |
|  | F2 -> AP-1/CREB/ELK/SRF/SP1 Expression Targets |
|  | HBEGF -> STAT Expression Targets |
|  | HBEGF -> FOXO3A Expression Target |
|  | HBEGF -> AP-1/ATF Expression Targets |
|  | HBEGF -> TP53 Expression Targets |
|  | HBEGF -> EP300/ETS/ETV/SP1 Expression Targets |
|  | HBEGF -> HIF1A Expression Targets |
|  | HBEGF -> MEF/MYOD Expression Target |
|  | HBEGF -> CREB/MYC Expression Targets |
|  | HGF -> AP-1/CREB/ELK/SRF/MYC Expression Targets |
|  | EGFR Transactivation in Cancer and Non-Cancer Cells |
|  | Receptors and Adaptor Proteins Activated in Cancer |
|  | Proteins with Altered Expression in Cancer-Associated Sustaining of Proliferative Signaling |
|  | Epithelial to Mesenchymal Transition in Cancer: Overview |
|  | Invadopodia Formation in Cancer Cells |
|  | IGF1 -> MEF/MYOD/MYOG Expression Targets |
|  | IGF1 -> STAT Expression Targets |
|  | IGF1 -> ELK/SRF/HIF1A/MYC/SREBF Expression Targets |
|  | Insulin -> STAT Expression Targets |
|  | Insulin -> CEBPA/CTNNB/FOXA/FOXO Expression Targets |
|  | Insulin -> MEF/MYOD Expression Targets |
|  | Insulin -> ELK/SRF/HIF1A/MYC/SREBF Expression Targets |
|  | Leptin -> STAT Expression Targets |
|  | Leptin -> ELK/SRF Expression Targets |
|  | GAST Expression Targets in GAST Expression Targets |
|  | CCL11 Expression Targets |
|  | EDN1 Expression Targets |
|  | Elevated Receptors -> Expression Targets in Endometrium |
|  | TGFB1-TGFBR1/AP-1 Expression Targets |
|  | TGFB1-ACVRL1 Expression Targets |
|  | IL1B Expression Targets |
|  | ROS in Angiotensin Mediated Cardiovascular Remodeling and Hypertrophy |
|  | prarg negative regulators, ps-positive |
|  | pprarg neg, uknown targets, ps-positive |
|  | PPARG negative regulators |
|  | pparg expr target\_regulators |
|  | Neighbors of disease exacerbation |
|  | Neighbors of tumor progression |
|  | Neighbors of cancer progression |
|  | Neighbors of overall survival |
|  | Neighbors of disease severity |
|  | Neighbors of tumor size |
|  | Neighbors of mortality |
|  | Neighbors of survival rate |
|  | Neighbors of infarct size |
|  | Neighbors of survival time |
|  | Neighbors of functional recovery |
|  | Neighbors of insulin sensitivity |
|  | Neighbors of bacterial load |
|  | Neighbors of cancer survival |
|  | Neighbors of lethality |
|  | Neighbors of tumor regression |
|  | Neighbors of disease-free survival |
|  | Neighbors of body weight |
|  | Neighbors of tumor promotion |
|  | Neighbors of neutrophil count |
|  | Neighbors of glucose tolerance |
|  | Neighbors of morbidity |
|  | Neighbors of blood pressure |
|  | Neighbors of leukocyte count |
|  | Neighbors of vascular leakage |
|  | Neighbors of vascular density |
|  | Neighbors of muscle mass |
|  | Neighbors of tumor aggressiveness |
|  | Neighbors of tumor resistance |
|  | Neighbors of blood glucose |
|  | Neighbors of glucose level |
|  | Neighbors of vascular tone |
|  | Neighbors of pregnancy outcome |
|  | Neighbors of fertility |
|  | Neighbors of comorbidity |
|  | Neighbors of fractional shortening |
|  | Neighbors of disease exacerbation |
|  | Neighbors of tumor progression |
|  | Neighbors of cancer progression |
|  | Neighbors of overall survival |
|  | Neighbors of disease severity |
|  | Neighbors of tumor size |
|  | Neighbors of mortality |
|  | Neighbors of survival rate |
|  | Neighbors of infarct size |
|  | Neighbors of survival time |
|  | Neighbors of functional recovery |
|  | Neighbors of insulin sensitivity |
|  | Neighbors of bacterial load |
|  | Neighbors of cancer survival |
|  | Neighbors of lethality |
|  | Neighbors of tumor regression |
|  | Neighbors of disease-free survival |
|  | Neighbors of body weight |
|  | Neighbors of tumor promotion |
|  | Neighbors of neutrophil count |
|  | Neighbors of glucose tolerance |
|  | Neighbors of morbidity |
|  | Neighbors of blood pressure |
|  | Neighbors of leukocyte count |
|  | Neighbors of vascular leakage |
|  | Neighbors of vascular density |
|  | Neighbors of muscle mass |
|  | Neighbors of tumor aggressiveness |
|  | Neighbors of tumor resistance |
|  | Neighbors of blood glucose |
|  | Neighbors of glucose level |
|  | Neighbors of vascular tone |
|  | Neighbors of pregnancy outcome |
|  | Neighbors of fertility |
|  | Neighbors of comorbidity |
|  | Neighbors of fractional shortening |
|  | Neighbors of cell development |
|  | Neighbors of immune response |
|  | Neighbors of cell population |
|  | Neighbors of inflammatory response |
|  | Neighbors of wound healing |
|  | Neighbors of angiogenesis |
|  | Neighbors of cell function |
|  | Neighbors of osteoclast development |
|  | Neighbors of epithelial to mesenchymal transition |
|  | Neighbors of cell proliferative response |
|  | Neighbors of chemotaxis |
|  | Neighbors of cell survival |
|  | Neighbors of osteoclast differentiation |
|  | Neighbors of cell phenotype |
|  | Neighbors of tissue remodeling |
|  | Neighbors of cell adhesion |
|  | Neighbors of T-cell activation |
|  | Neighbors of endothelial cell proliferation |
|  | Neighbors of fibrogenesis |
|  | Neighbors of tumor growth |
|  | Neighbors of tissue repair |
|  | Neighbors of cell count |
|  | Neighbors of cell infiltration |
|  | Neighbors of cell interaction |
|  | Neighbors of SMC proliferation |
|  | Neighbors of lumen formation |
|  | Neighbors of endothelial cell migration |
|  | Neighbors of adipogenesis |
|  | Neighbors of lipid storage |
|  | Neighbors of cell damage |
|  | Neighbors of pregnancy |
|  | Neighbors of aging |
|  | Neighbors of cell motility |
|  | Neighbors of monocyte migration |
|  | Neighbors of fibroblast proliferation |
|  | Neighbors of adipocyte differentiation |
|  | Neighbors of microglial activation |
|  | Neighbors of regeneration |
|  | Neighbors of cell migration |
|  | Neighbors of cancer growth |
|  | Neighbors of stem cell proliferation |
|  | Neighbors of smooth muscle cell migration |
|  | Neighbors of hepatic regeneration |
|  | Neighbors of transendothelial migration |
|  | Neighbors of leukocyte migration |
|  | Neighbors of cell invasion |
|  | Neighbors of heart function |
|  | Neighbors of lung development |
|  | Neighbors of transcription activation |
|  | Neighbors of hepatocyte proliferation |
|  | Neighbors of cell transdifferentiation |
|  | Neighbors of neuroprotection |
|  | Neighbors of stem cell differentiation |
|  | Neighbors of neuronal death |
|  | Neighbors of cell growth |
|  | Neighbors of ROS generation |
|  | Neighbors of epithelial cell proliferation |
|  | Neighbors of first trimester pregnancy |
|  | Neighbors of cancer cell growth |
|  | Neighbors of hemato-encephalic barrier |
|  | Neighbors of colony formation |
|  | Neighbors of cell differentiation |
|  | Neighbors of keratinocyte proliferation |
|  | Neighbors of stem cell migration |
|  | Neighbors of cell homeostasis |
|  | Neighbors of psoriasis |
|  | Neighbors of atherosclerosis |
|  | Neighbors of fibrosis |
|  | Neighbors of colitis |
|  | Neighbors of injury |
|  | Neighbors of atherogenesis |
|  | Neighbors of vascular remodeling |
|  | Neighbors of metastasis |
|  | Neighbors of carcinogenesis |
|  | Neighbors of inflammation |
|  | Neighbors of pathological angiogenesis |
|  | Neighbors of obesity |
|  | Neighbors of vascular disease |
|  | Neighbors of diabetes mellitus |
|  | Neighbors of asthma |
|  | Neighbors of pneumonia |
|  | Neighbors of hyperplasia |
|  | Neighbors of liver fibrosis |
|  | Neighbors of chronic obstructive pulmonary disease |
|  | Neighbors of myocardial infarction |
|  | Neighbors of reperfusion injury |
|  | Neighbors of insulin resistance |
|  | Neighbors of infection |
|  | Neighbors of vascular injury |
|  | Neighbors of renal injury |
|  | Neighbors of preeclampsia |
|  | Neighbors of macrophage infiltration |
|  | Neighbors of liver injury |
|  | Neighbors of death |
|  | Neighbors of neutrophil infiltration |
|  | Neighbors of heart disease |
|  | Neighbors of diabetic nephropathy |
|  | Neighbors of systemic sclerosis |
|  | Neighbors of hepatocellular carcinoma |
|  | Neighbors of acute lung injury |
|  | Neighbors of gastric cancer |
|  | Neighbors of atherosclerotic plaque |
|  | Neighbors of cardiac remodeling |
|  | Neighbors of breast cancer |
|  | Neighbors of ischemia |
|  | Neighbors of interstitial fibrosis |
|  | Neighbors of neoplasm invasion |
|  | Neighbors of heart failure |
|  | Neighbors of lung disease |
|  | Neighbors of pulmonary fibrosis |
|  | Neighbors of cardiovascular disease |
|  | Neighbors of stroke |
|  | Neighbors of cancer |
|  | Neighbors of cerebral ischemia |
|  | Neighbors of type 2 diabetes |
|  | Neighbors of neoplasm |
|  | Neighbors of myocardial fibrosis |
|  | Neighbors of liver disease |
|  | Neighbors of hypertrophy |
|  | Neighbors of ovarian cancer |
|  | Neighbors of hypertension |
|  | Neighbors of proteinuria |
|  | Neighbors of epithelial cell |
|  | Neighbors of keratinocyte |
|  | Neighbors of vascular smooth muscle cell |
|  | Neighbors of monocyte |
|  | Neighbors of endometrial stromal cell |
|  | Neighbors of macrophage |
|  | Neighbors of adipocyte |
|  | Neighbors of activated monocyte |
|  | Neighbors of fibroblast |
|  | Neighbors of airway epithelial cell |
|  | Neighbors of stromal cell |
|  | Neighbors of trophoblast |
|  | Neighbors of intestine epithelium cell |
|  | Neighbors of hepatic stellate cell |
|  | Neighbors of hepatocyte |
|  | Neighbors of kidney mesangial cell |
|  | Neighbors of endothelial cell |
|  | Neighbors of dendritic cell |
|  | Neighbors of Kuppfer cell |
|  | Neighbors of kidney tubule cell |
|  | Neighbors of smooth muscle myocyte |
|  | Neighbors of astrocyte |
|  | Neighbors of lung fibroblast |
|  | Neighbors of mesothelial cell |
|  | Neighbors of mononuclear phagocyte |
|  | Neighbors of capillary endothelial cell |
|  | Neighbors of granulocyte |
|  | Neighbors of cardiac myocyte |
|  | Neighbors of cancer-associated fibroblast |
|  | Neighbors of corneal epithelial cell |
|  | Neighbors of stem cell |
|  | Neighbors of tumor infiltrating macrophage |
|  | Neighbors of sinusoidal cell |
|  | Neighbors of chondrocyte |
|  | Neighbors of insulin-secreting cell |
|  | Neighbors of mesenchymal cell |
|  | Neighbors of T-cell |
|  | Neighbors of myofibroblast |
|  | Neighbors of mast cell |
|  | Neighbors of neuroglia |
|  | Neighbors of aortic smooth muscle cell |
|  | Neighbors of podocyte |
|  | Neighbors of pericyte |
|  | Neighbors of epidermis cell |
|  | Neighbors of kidney cell |
|  | Neighbors of M2 macrophage |
|  | Neighbors of lung |
|  | Neighbors of kidney |
|  | Neighbors of intestine |
|  | Neighbors of blood vessel |
|  | Neighbors of gastrointestinal tract |
|  | Neighbors of placenta |
|  | Neighbors of retina |
|  | Neighbors of blood vessel wall |
|  | Neighbors of stomach |
|  | Neighbors of central nervous system |
|  | Neighbors of kidney glomerulus |
|  | Neighbors of glands |
|  | Neighbors of eye |
|  | Neighbors of cornea |
|  | Neighbors of cerebral cortex |
|  | Neighbors of bladder |
|  | Neighbors of nervous system |
|  | Neighbors of blastocyst |
|  | Neighbors of NF-kB family |
|  | Neighbors of oxidized LDL |
|  | Neighbors of PPAR |
|  | Neighbors of Jun/Fos |
|  | Neighbors of PKC |
|  | Neighbors of mitogen-activated protein kinase |
|  | Neighbors of transforming growth factor |
|  | Neighbors of cytokine |
|  | Neighbors of PDGF |
|  | Neighbors of Ras GTPase |
|  | Neighbors of growth factor |
|  | Neighbors of PKA |
|  | Neighbors of LDL |
|  | Neighbors of protein tyrosine kinase |
|  | Neighbors of IgG |
|  | Neighbors of p38 |
|  | Neighbors of T-cell receptor |
|  | Neighbors of histone deacetylase |
|  | Neighbors of PDGF-BB |
|  | Neighbors of GPCR |
|  | Neighbors of Notch |
|  | Neighbors of aldehyde reductase |
|  | Neighbors of mitogen-activated protein kinase kinase |
|  | Neighbors of Ang II receptor |
|  | Neighbors of endothelin |
|  | Neighbors of NOS |
|  | Neighbors of proteasome endopeptidase complex |
|  | Neighbors of non-coding RNA |
|  | Neighbors of alpha2 adrenoceptor |
|  | Neighbors of phosphoinositide phospholipase C |
|  | 2\_Interleukin-17 and interleukin-22 signaling in psoriasis |
|  | Interleukin-17 and interleukin-22 signaling in psoriasis |

---

|  |  |
| --- | --- |
| Group | Chemokines, Growth Factors and Receptors Involved in Cancer Cells Chemotaxis |

---

|  |  |
| --- | --- |
| MedScan ID | 1839 |

---

|  |  |
| --- | --- |
| LocusLink ID | 1839 |
|  | 15200 |
|  | 25433 |

---

|  |  |
| --- | --- |
| Alias | DTSF |
|  | LOC225370 |
|  | proheparin-binding EGF-like growth factor |
|  | heparin-binding epidermal growth-like growth factor |
|  | GFHB |
|  | DTS |
|  | heparin-binding epidermal growth factor |
|  | AW047313 |
|  | pro-HB-EGF |
|  | pro-heparin-binding EGF-like growth factor |
|  | HBGF\_HUMAN |
|  | diphtheria toxin receptor (heparin-binding epidermal growth factor-like growth factor) |
|  | heparin-binding EGF-like growth factors |
|  | DTR |
|  | heparin-binding epidermal growth factor-like growth factor |
|  | HB-EGF |
|  | diphtheria toxin receptor |
|  | HEGFL |
|  | HBEGF |
|  | proHB-EGF |
|  | heparin-binding EGF-like growth factor |
|  | diphtheria toxin receptors |
|  | OTTHUMP00000223860 |
|  | MGC107656 |
|  | Diphtheria toxin receptor (heparin-binding EGF-like growth factor) |
|  | heparin binding epidermal growth factor-like growth factor |
|  | Diphtheria toxin receptor (heparin binding epidermal growth factor - like growth factor) |
|  | heparin-binding epidermal -growth - like growth factor |
|  | heparin-binding epidermal -growth factor |
|  | Diphteria toxin receptors |
|  | Diphteria toxin receptor |

---

|  |  |
| --- | --- |
| GO ID | 0005154 |
|  | 0008083 |
|  | 0008201 |
|  | 0038128 |
|  | 0000165 |
|  | 0060326 |
|  | 0007173 |
|  | 0061024 |
|  | 0007517 |
|  | 0051545 |
|  | 0042059 |
|  | 0030307 |
|  | 0030335 |
|  | 0008284 |
|  | 0051549 |
|  | 0050731 |
|  | 0051897 |
|  | 0048661 |
|  | 0090303 |
|  | 2000145 |
|  | 0008016 |
|  | 0007165 |
|  | 0035313 |
|  | 0009986 |
|  | 0030669 |
|  | 0030665 |
|  | 0030666 |
|  | 0005576 |
|  | 0005615 |
|  | 0005887 |
|  | 0005886 |
|  | 0001525 |
|  | 0001832 |
|  | 0016477 |
|  | 0016021 |
|  | 0016020 |
|  | 0007399 |
|  | 0010469 |
|  | 0005088 |
|  | 0046934 |
|  | 0004713 |
|  | 0018108 |
|  | 0046854 |
|  | 0048015 |
|  | 0043547 |
|  | 0014066 |
|  | 0038095 |
|  | 0008543 |
|  | 0045087 |
|  | 0048011 |
|  | 0009405 |

---

|  |  |
| --- | --- |
| KEGG ID | hsa:1839 |
|  | mmu:15200 |
|  | rno:25433 |

---

|  |  |
| --- | --- |
| Organism | Homo sapiens {Organism urn:agi-taxid:9606} |
|  | Mus musculus {Organism urn:agi-taxid:10090} |
|  | Rattus norvegicus {Organism urn:agi-taxid:10116} |
|  | Homo sapiens |
|  | Mus musculus |
|  | Rattus norvegicus |

---

|  |  |
| --- | --- |
| Mouse chromosome position | 18 19.46 cM |

---

|  |  |
| --- | --- |
| OMIM ID | 126150 |

---

|  |  |
| --- | --- |
| Rat chromosome position | 18p11 |

---

|  |  |
| --- | --- |
| Hugo ID | 3059 |
|  | HGNC:3059 |

---

|  |  |
| --- | --- |
| Human chromosome position | 5q31.3 |
|  | 5q23 |

---

|  |  |
| --- | --- |
| Swiss-Prot Accession | Q99075 |
|  | Q99075.1 |
|  | Q06186 |
|  | Q5FW64 |
|  | Q06186.1 |
|  | Q06175 |
|  | Q06175.1 |
|  | B2R821 |

---

|  |  |
| --- | --- |
| PIR ID | A38432 |
|  | JC1410 |
|  | JC1409 |

---

|  |  |
| --- | --- |
| GenBank ID | NC\_000005 |
|  | NM\_001945 |
|  | NP\_001936 |
|  | AC004634 |
|  | AAC15470 |
|  | AY164533 |
|  | AAN46738 |
|  | CH471062 |
|  | EAW62069 |
|  | GM880654 |
|  | CAV28318 |
|  | L17029 |
|  | AAA50562 |
|  | L17030 |
|  | AAA50563 |
|  | L17031 |
|  | AAA50564 |
|  | AK222688 |
|  | BAD96408 |
|  | AK307310 |
|  | AK313202 |
|  | BAG36018 |
|  | BC033097 |
|  | AAH33097 |
|  | D55114 |
|  | M60278 |
|  | AAA35956 |
|  | Q99075 |
|  | NC\_000084 |
|  | NM\_010415 |
|  | NP\_034545 |
|  | AC147220 |
|  | AH006539 |
|  | AAC42069 |
|  | AH006744 |
|  | AAC52617 |
|  | CH466557 |
|  | EDK97159 |
|  | AK052084 |
|  | AK145548 |
|  | BAE26501 |
|  | AK160575 |
|  | BAE35883 |
|  | AK164309 |
|  | BAE37729 |
|  | AW489543 |
|  | BC089607 |
|  | AAH89607 |
|  | L07264 |
|  | AAA37542 |
|  | Q06186 |
|  | NC\_005117 |
|  | NM\_012945 |
|  | NP\_037077 |
|  | AC\_000086 |
|  | AAHX01094545 |
|  | AAHX01094546 |
|  | AAHX01094547 |
|  | AC097756 |
|  | CH473974 |
|  | EDL76294 |
|  | L05489 |
|  | AAA81780 |
|  | Q06175 |
|  | NC\_018916 |
|  | AMYH02012330 |
|  | EU832647 |
|  | ACE87738 |
|  | EU832724 |
|  | ACE87056 |
|  | AC\_000040 |
|  | AAHY01137750 |
|  | L36024 |
|  | L36025 |
|  | L36026 |
|  | L36027 |
|  | U39189 |
|  | U39190 |
|  | U39191 |
|  | U39192 |
|  | AC\_000137 |
|  | ABBA01026434 |

---

|  |  |
| --- | --- |
| Swiss-Prot ID | HBEGF\_HUMAN |
|  | HBEGF\_MOUSE |
|  | HBEGF\_RAT |

---

|  |  |
| --- | --- |
| Cell Localization | Secreted |
|  | Extracellular space |
|  | Cell membrane |
|  | Secreted, extracellular space |

---

|  |  |
| --- | --- |
| Ensembl ID | ENSG00000113070 |
|  | ENSP00000230990.6 |
|  | ENST00000230990.7 |
|  | ENSMUSG00000024486 |
|  | ENSMUSP00000025363.5 |
|  | ENSMUST00000025363.6 |
|  | ENSRNOG00000018646 |
|  | ENSRNOP00000025157.5 |
|  | ENSRNOT00000025157.6 |
|  | ENSP00000230990 |
|  | ENST00000230990 |
|  | ENSMUSP00000025363 |
|  | ENSMUST00000025363 |
|  | ENSRNOP00000025157 |
|  | ENSRNOT00000025157 |

---

|  |  |
| --- | --- |
| MGI ID | MGI:96070 |

---

|  |  |
| --- | --- |
| RGD ID | 2526 |

---

|  |  |
| --- | --- |
| Unigene ID | Hs.799 |
|  | Hs.592942 |
|  | Mm.289681 |
|  | Rn.10148 |

---

|  |  |
| --- | --- |
| Homologene ID | 1466 |

---

|  |  |
| --- | --- |
| Shape | Rhomb |

---
